# Fatty-binding protein and galectin of *Baylisascaris schroederi*: Prokaryotic expression and preliminary evaluation of serodiagnostic potential

**DOI:** 10.1371/journal.pone.0182094

**Published:** 2017-07-27

**Authors:** Ying Sun, Yu Li, Yiran Wu, Lang Xiong, Caiwu Li, Chengdong Wang, Desheng Li, Jingchao Lan, Zhihe Zhang, Bo Jing, Xiaobing Gu, Yue Xie, Weimin Lai, Xuerong Peng, Guangyou Yang

**Affiliations:** 1 Department of Parasitology, College of Veterinary Medicine, Sichuan Agricultural University, Chengdu, China; 2 China Conservation and Research Center for Giant Panda, Wolong, China; 3 Chengdu Research Base of Giant Panda Breeding, Chengdu, China; 4 College of Science, Sichuan Agricultural University, Ya’an, China; Universidad Nacional de la Plata, ARGENTINA

## Abstract

*Baylisascaris schroederi* is a common parasite of captive giant pandas. The diagnosis of this ascariasis is normally carried out by a sedimentation-floatation method or PCR to detect eggs in feces, but neither method is suitable for early diagnosis. Fatty acid-binding protein (FABP) and galectin (GAL) exist in various animals and participate in important biology of parasites. Because of their good immunogenicity, they are seen as potential antigens for the diagnosis of parasitic diseases. In this study, we cloned and expressed recombinant FABP and GAL from *B*. *schroederi* (rBs-FABP and rBs-GAL) and developed indirect enzyme-linked immunosorbent assays (ELISAs) to evaluate their potential for diagnosing ascariasis in giant pandas. Immunolocalization showed that Bs-FABP and Bs-GAL were widely distributed in adult worms. The ELISA based on rBs-FABP showed sensitivity of 95.8% (23/24) and specificity of 100% (12/12), and that based on rBs-GAL had sensitivity of 91.7% (22/24) and specificity of 100% (12/12).

## Introduction

Giant panda (*Ailuropoda melanoleuca*), known as a ‘‘living fossil” with a history of about 7 million years [1], is a national first class protected animal and endemic to China. According to the results from the Fourth National Survey on Giant Pandas from 2011 to 2014, conducted by the State Forestry Administration, as of the end of 2013, there were about 1864 wild pandas [2], mainly distributed in mountain areas of Sichuan, Shaanxi and Gansu Provinces [3]. The 2016 Annual Conference of the Chinese Committee of Breeding Techniques for Giant Pandas noted that 469 captive giant pandas are present in 84 institutions in 18 countries and regions (unpublished data).

*Baylisascaris schroederi* is the most important parasite of captive giant pandas. A report showed that the positive rate of *B*. *schroederi* eggs in all tested samples from captive pandas was 25.71% (54/210) [4]. The adult worms usually parasitize the small intestine, and are occasionally found in the mouth, pharynx, trachea, stomach, and so on [5, 6]. On infection with *B*. *schroederi*, the pandas suffer depression, anorexia, emaciation, and even death [7]. Methods like PCR, including amplification of the mitochondrial cytochrome *c* oxidase subunit II (COII) gene (331 bp and 279 bp fragments) and mitochondrial 12S rRNA [8–10], or sedimentation-floatation assays, can be used to detect *B*. *schroederi* eggs in feces of infected giant pandas. However, after eggs containing L_2_ larvae hatch in the host, it takes 77–93 days before they mature and begin to ovulate [11]. Thus, the currently available methods are unsuitable for early detection of *B*. *schroederi* since the larvae may be in their migratory period or not yet have matured in the intestine. Because of the frequent exchange of captive pandas in various regions and countries and quarantine requirements, it is important to establish a method for rapid and accurate early diagnosis of *B*. *schroederi*.

Fatty acid-binding proteins (FABPs) belong to a large family of intracellular lipid-binding proteins; these low-molecular-weight proteins are distributed widely in tissues from bacteria to mammals [12]. Despite great differences in primary structures, FABPs share similar tertiary structures and functions of transporting lipids [13], and could play an important role in helminths [14]. Galectins (GAL), also named β-galactoside binding proteins [15], are members of the lectin family. They are conserved in evolution [16] and can participate in numerous biological functions [17], including internal stability, cell adhesion, apoptosis, and others. Galectins have a highly-specific affinity for β-galactoside and can excite host immune response during parasitic infection [18]. Both GALs and FABPs are considered to be latent antigens for vaccines or diagnosis due to their good immunogenicity. For instance, a 14.5-kDa FABP of *Fasciola gigantica* has been used as a diagnostic antigen for human fascioliasis [19–21].

The aim of this study was to clone and express *B*. *schroederi* FABP and GAL beginning with transcriptome data. Immunoblotting and immunolocalization were performed using the purified proteins. Indirect enzyme-linked immunosorbent assays (iELISAs) based on recombinant *B*. *schroederi* FABP (rBs-FABP) and GAL (rBs-GAL) were established to show the diagnostic value of these proteins, and to provide a reference for further research and development of early detection assays for *B*. *schroederi* infection in giant pandas.

## Materials and methods

### Parasites and animals

Adult *B*. *schroederi* derived from the feces of giant pandas were stored and provided by the Department of Parasitology, College of Veterinary Medicine, Sichuan Agricultural University. Four male New Zealand White rabbits (1.4–2.2 kg) were obtained from the Laboratory Animal Center of Sichuan Agricultural University and 34 female ICR mice (specific-pathogen-free grade) of 6- to 8-weeks-old were purchased from Chengdu Dashuo Animal Experimental Center. All procedures were carried out in strict accordance with the Guide for the Care and Use of Laboratory Animals of the Animal Ethics Committee of Sichuan Agricultural University (Chengdu, China) (Approval No. 2016–030). All ethical efforts were made to minimize suffering.

### Sera

Twenty-four positive serum samples were isolated from *B*. *schroederi*-naturally infected giant pandas in Wolong Giant Panda Conservation and Research Center. The vomit and feces of the pandas were monitored for a long time for the presence of *B*. *schroederi* and eggs were detected in the feces by traditional sedimentation-floatation assay. Thirty-six negative serum samples were obtained from *B*. *schroederi*-free giant pandas in Chengdu Research Base of Giant Panda Breeding. No adult worms or *B*. *schroederi* eggs were found in these pandas for 3–4 months. All sera were collected in routine medical examinations of giant pandas, and the welfare of animals was considered during collection. All samples were stored at −20°C until use.

### Cloning, expression and purification of recombinant proteins

Total RNA was extracted from adult *B*. *schroederi* stored in liquid nitrogen using a RNA-prep Pure Tissue Kit (Tiangen, Beijing, China), and first strand cDNA was transcribed using the Reverse Transcription System Kit (Thermo, USA) according to the manufacturer's protocols. Based on the assembled and annotated genome and transcriptome datasets of *B*. *schroederi* (unpublished data), the cDNA sequences of *B*. *schroederi* FABP (asmbl_65323) and GAL (asmbl_32016) were identified. Primers for PCR were designed:

FABP-F (5′-CGC***GGATCC***ATGTCGGAAGCGTTCATTGG-3′, *Bam*HI)

FABP-R (5′-CCC**AAGCTT**CTATCTCTTCGAGTGCAGACGTTT-3′, *Hin*dIII)

GAL-F (5′-CGC**GGATCC**ATGCCTGTCACTTTTG-3′, *Bam*HI)

GAL-R (5′-CCC**AAGCTT**TCAAACACCATAATGAACT-3′, *Hin*dIII)

The PCR products were separated by agarose gel electrophoresis and recovered using a DNA Gel Extraction Kit (Tiangen). Then, the pure DNA fragments were respectively cloned into vector pMD19-T (TaKaRa, Dalian, China) and sequenced. After digestion with restriction enzymes *Bam*HI and *Hin*dIII and ligation into the expression vector pET32a(+) (Novagen, Madison, USA), the correct, sequenced plasmids were transformed into *Escherichia coli* BL21 (DE3) cells (Invitrogen, Carlsbad, USA), which were cultivated and induced for protein expression with 1 mM isopropyl-β-D-thiogalactopyranoside at 37°C. The induced bacterial cells were centrifuged and ultrasonic disruption was applied. The recombinant proteins were purified by Ni^2+^-affinity chromatography (Bio-Rad, CA, USA) according to the manufacturer’s protocol and separated and characterized by sodium dodecyl sulfate-polyacrylamide gel electrophoresis (SDS-PAGE). A micro-BCA protein assay kit (NJJCBIO, China) was applied for determining the concentration of purified proteins.

### Sequence analysis

The translation of cDNA to amino acid sequences was performed using the Open Reading Frame Finder (http://www.ncbi.nlm.nih.gov/gorf/gorf.html) and DNASTAR software (Madison, WI, USA). Multiple sequence alignment was performed and a phylogenetic tree constructed using ClustalX (version 1.83) and MEGA (version 5.05) software using the neighbor-joining (NJ) method. The molecular weight (Mw) of proteins was calculated using the Compute pI/Mw tool (http://web.expasy.org/compute_pi/). Signal sequences were predicted by the Compute SignalP 4.0 server (http://www.cbs.dtu.dk/services/SignalP/).

### Preparation of polyclonal antibodies

Two male New Zealand White rabbits were subcutaneously immunized with 200 μg purified rBs-FABP and two with rBs-GAL, each protein mixed with isopycnic Freund's adjuvant (Sigma-Aldrich, USA). Immunizations were performed at intervals of 2 weeks. Two weeks after the fourth immunization, serum was isolated. At the same time, two naïve rabbits (the control group) were also bled for serum. The isolated serum was purified on a Protein A affinity chromatography column (Bio-Rad) to obtain polyclonal antibodies, i.e., rBs-FABP-IgG and rBs-GAL-IgG.

### Immunoblotting

After SDS-PAGE (12%), recombinant proteins were transferred to nitrocellulose (NC) membranes (0.2 μm, Bio-Rad). The NC sheets were blocked in 5% skim milk in Tris-buffered saline (TBS) for 2 h at room temperature. After three washes using TBS with Tween-20 (TBST), the membranes were incubated with the serum from naturally infected giant pandas diluted by TBS (1:100) overnight at 4°C. At the same time, negative controls were performed with serum from *B*. *schroederi*-free giant pandas. Following washing steps, horseradish peroxidase (HRP)-conjugated rabbit anti-panda IgG (Zen-bioscience Co., Chengdu, China) at a dilution of 1:2000 was applied to the membranes for 2 h at room temperature. The signals were revealed using an Enhanced HRP-DAB Chromogenic Substrate Kit (Tiangen).

Total proteins of *B*. *schroederi* worms were extracted in an NP-40 cell lysis buffer (Boster, Wuhan, China). All procedures for analyzing total proteins were the same as above, except the serum was replaced by purified rBs-FABP-IgG or rBs-GAL-IgG (or naïve rabbit serum), and the secondary antibody was replaced by HRP-conjugated goat anti-rabbit IgG (Boster).

### Immunolocalization of proteins in *B*. *schroederi* adult worms

Adult female *B*. *schroederi* were placed in paraffin blocks and sliced into sections of 5-μm thickness. The paraffin sections were prepared after a series of procedures including toasting, dewaxing, hydration and heat repair, then blocked with normal goat serum (Boster) for 20 min at room temperature. Then, purified rBs-FABP-IgG and rBs-GAL-IgG (at a dilution of 1:100 in phosphate-buffered saline [PBS]) were respectively added to the slices, which were later kept in a wet box at 4°C overnight. After three washes, the slices were incubated with fluorescein isothiocyanate-conjugated goat anti-rabbit IgG (Boster) at a dilution of 1:100 in 1% Evans Blue (Leagene Biotechnology, Beijing, China) at room temperature for 20 min in darkness. After washing, fluorescence dying and sealing with glycerol carbonic acid buffer, the slices were examined and imaged under a fluorescence microscope (Nikon, Tokyo, Japan).

### Development of indirect ELISAs

rBs-FABP and rBs-GAL proteins were diluted in 0.1 M carbonate buffer (pH 9.6) with the concentration varying from 4 μg/well to 0.03 μg/well and 6 μg/well to 0.05 μg/well respectively. Protein samples were added into 96-well ELISA plates and kept at 4°C overnight. The plates were washed three times with 0.01 M PBS containing 0.05% Tween-20 (PBST) for 5 min per wash and then blocked with 5% skim milk (100 μL/well) at 37°C for 1 h. Negative and positive sera from giant pandas were diluted in PBS to 1:20, 1:40, 1:80, 1:160, 1:320 and 1:640, then added to the wells (100 μL/well) and incubated at 37°C for 1 h. Then, HRP-labeled rabbit anti-panda IgG diluted 1:4000 in PBS was added into the plates (100 μL/well) for another 1 h at 37°C. Finally, tetramethylbenzidine (TMB; Tiangen) was applied in the dark for 15 min and the color development process was stopped by the addition of 2 M H_2_SO_4_. Optical density (OD) of all wells was measured at 450 nm. The well which had the maximum difference in OD_450_ value between positive and negative serum (P/N), and for which the positive OD_450_ value was closest to 1.0, was chosen as the optimal working conditions. In these conditions, 24 negative serum samples were examined to determine the cut-off value following the formula: mean (MN) OD_450_ value from the negative sera + 3 standard deviations (SD).

### Intra- and interassay variability

Two groups of tests were set up; five positive sera from *B*. *schroederi*-infected giant pandas were used and each had three repeats in one coated 96-well ELISA plate as the intra-assay variability group; the same samples as in the former group were applied continuously to another three coated plates as the interassay variability group. Both groups were assessed in the optimized conditions identified above. After measuring the OD_450_ of all samples, the coefficient of variation (CV) was calculated according to the formula: SD/MN ×100%.

### Sensitivity and specificity

Twenty-four serum samples from *B*. *schroederi* positive pandas were tested by iELISA. Sensitivity was determined according to the criteria: Sensitivity = positive samples by ELISA/true positive number × 100%. Twelve negative panda serum samples were also tested. Specificity was determined according to the criteria: Specificity = number of negative samples by ELISA/true negative number × 100%.

### Indirect ELISA of samples from mice

Eggs freshly collected from infected-panda feces were placed in a Petri dish containing 2.5% potassium dichromate solution and incubated in a constant temperature incubator at 28°C. Four weeks later, infective *B*. *schroederi* embryonated eggs were obtained. Ten mice were artificially infected by gavage, each with 1,000 eggs. At the same time, a control group of 24 mice were set. Blood was collected after 2 weeks, and serum was separated and stored at −20°C. Serum titer of infected mice was detected by agar diffusion test. A 1% agarose solution was prepared in 0.01 M PBS, poured into a plate, and holes were punched with an agar diffusion test hole puncher. Total proteins of *B*. *schroederi* were added as antigen in the central well, then diluted positive sera and negative sera from mice were added into surrounding wells. The plate was placed at 37°C for 24 h to detect the precipitation line.

In the optimal conditions of the established ELISAs (other than changing the secondary antibody to HRP-labeled goat anti-mouse), 24 negative mouse serum samples were selected to assess the cut-off value, and 10 positive mouse serum samples were chosen to determine the assay sensitivity.

## Results

### Cloning, sequencing and expression of Bs-FABP and Bs-GAL

Fragments of 438 bp (FABP) and 429 bp (GAL) were amplified from the total RNA of *B*. *schroederi* and identified through BLAST analysis. The coding sequences of the genes encoded 145 and 142 amino acids respectively, with predicted protein molecular weights of 16.7 kDa and 16.1 kDa. Bioinformatics analysis software predicted no signal peptide or transmembrane region in either FABP and GAL; both were characterized as a soluble protein.

**Fig 1:**
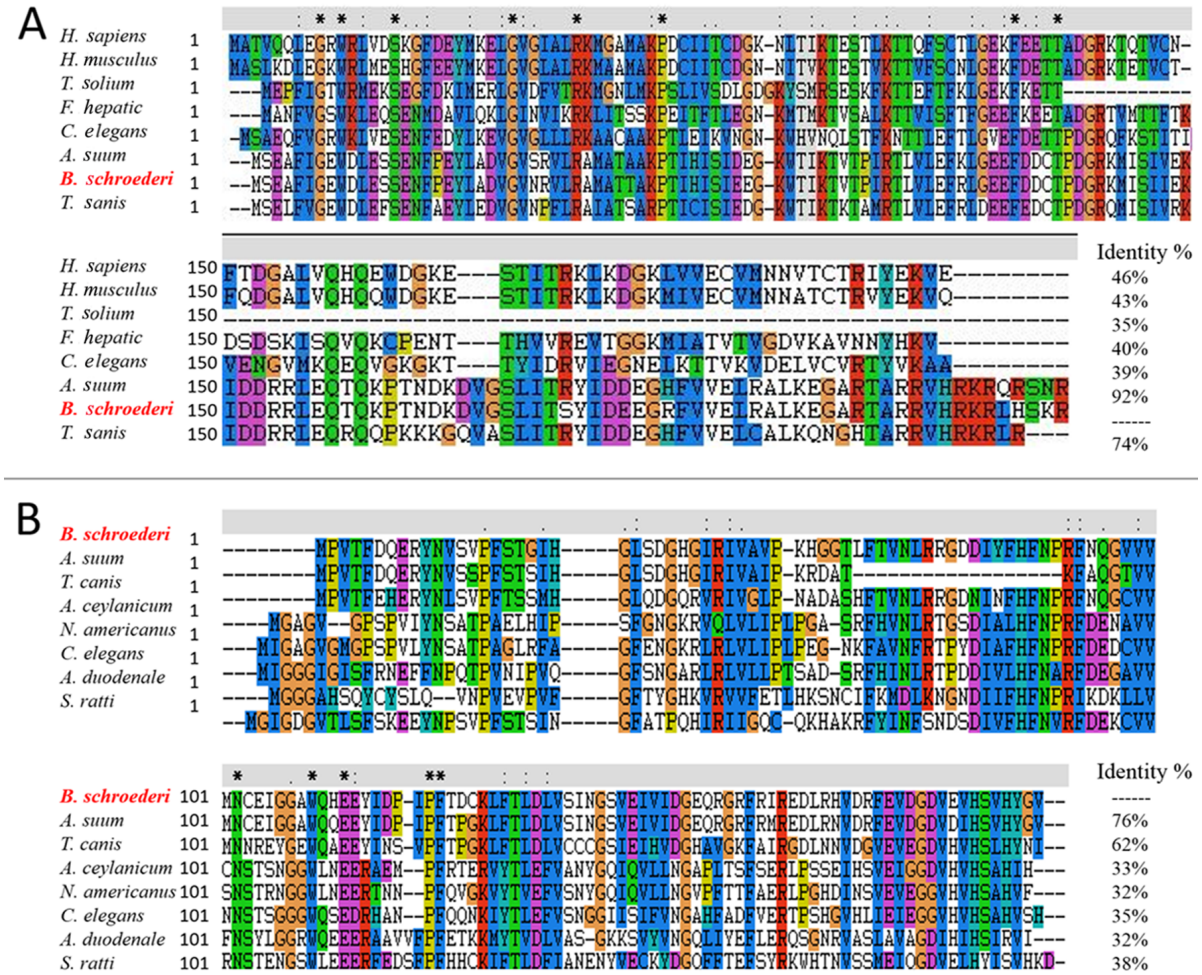
Multiple sequence alignment of fatty acid-binding protein (FABP) and galectin (GAL) in *Baylisascaris schroederi* and other species. A: The following sequences of FABP were retrieved from the NCBI protein database and aligned using Clustal X1.83 software: *A*. *suum* (ERG79338.1), *T*. *canis* (KHN74594.1), *C*. *elegans* (NP_491928.1), *F*. *hepatica* (CAB65015.1), *T*. *solium* (ABB76135.1), *H*. *sapiens* (NP_001435.1), *M*. *musculus* (AAI00544.1). B: The following sequences of GAL were retrieved from the NCBI protein database: *A*. *suum* (ERG81589.1), *T*. *canis* (KHN87849.1), *C*. *elegans* (NP_497215.1), *A*. *ceylanicum* (EYC36147.1), *N*. *americanus* (XP_013299521.1), *S*. *ratti* (CEF66290.1), *A*. *duodenale* (KIH48405.1). Residue identity (*), strong similarity (:) and weak similarity (.) are indicated.

**Fig 2:**
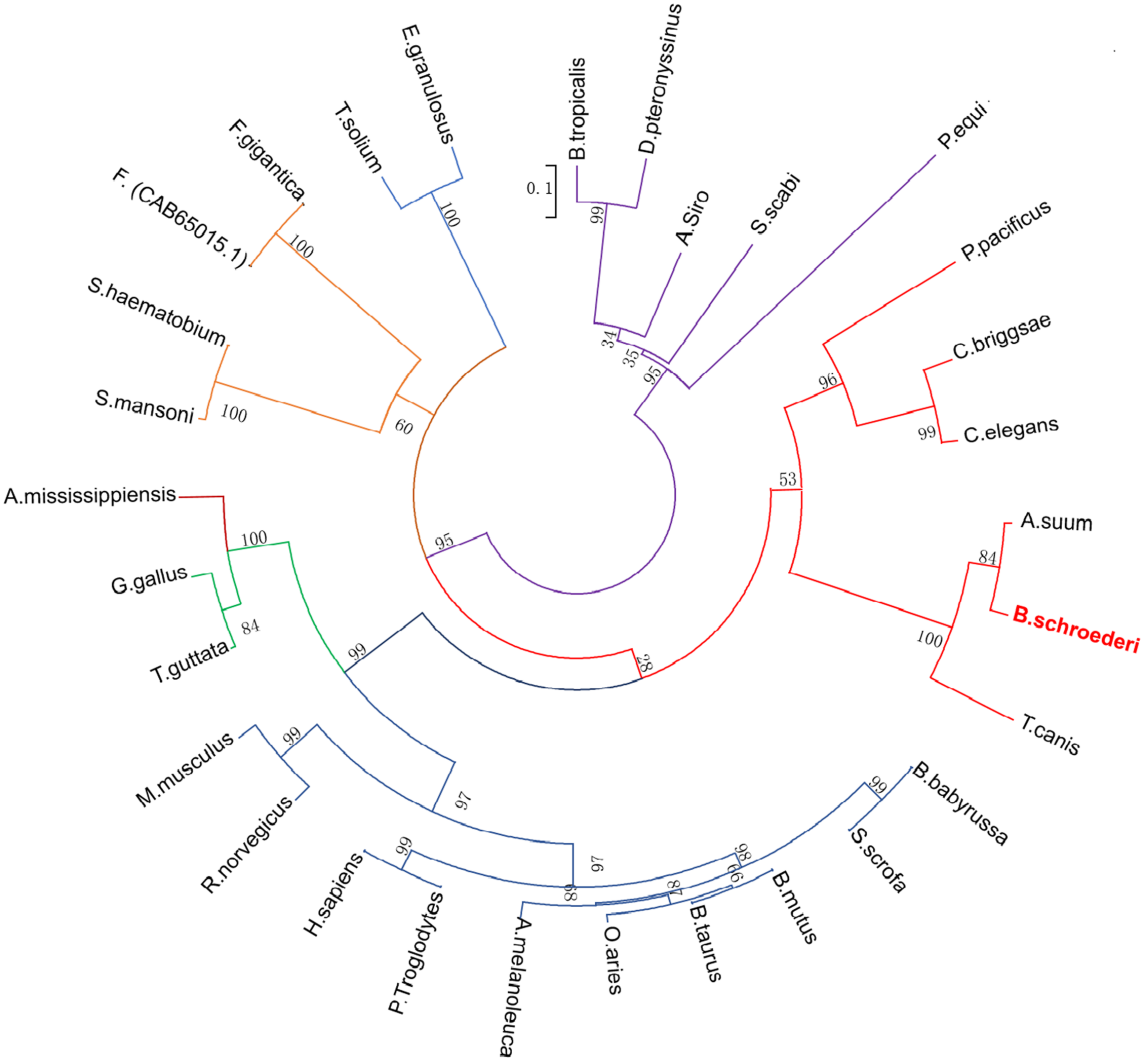
Phylogenetic analysis of FABP. An unrooted phylogenetic tree was inferred by neighbor-joining (NJ) analysis. The scale bar indicates evolutionary distance (0.1 substitutions per position). Nodal support is given in percent (1000 bootstrap replicates). The protein sequences used in the tree are from: *P*. *pacificus* (KKA71402.1), *C*. *briggsae* (XP002640204.1), *C*. *elegans* (NP_491928.1), *A*. *suum* (ERG79338.1), *T*. *canis* (KHN74594.1), *B*. *babyrussa* (ABY84518.1), *S*. *scrofa* (AAX14717.1), *B*. *mutus* (AJE26250.1), *B*. *taurus* (NP_776740.1), *O*. *aries* (ACM43302.1), *A*. *melanoleuca* (AFJ19861.1), *P*. *troglodytes* (JAA33798.1), *H*. *sapiens* (NP_001435.1), *R*. *norvegicus* (EDM00998.1), *M*. *musculus* (AAI00544.1), *T*. *guttata* (ACH43723.1), *G*. *gallus* (NP_001006346.1), *A*. *mississippiensis* (KYO25921.1), *S*. *mansoni* (AAA63516.1), *S*. *haematobium* (BAF62288.1), *F*. *hepatica* (CAB65015.1), *F*. *gigantica* (AHA90589.1), *T*. *solium* (ABB76135.1), *E*. *granulosus* (AAK00579.1), *B*. *tropicalis* (AAP35071.1), *D*. *pteronyssinus* (ADK92390.1), and *A*. *siro* (ABL09307.1).

**Fig 3:**
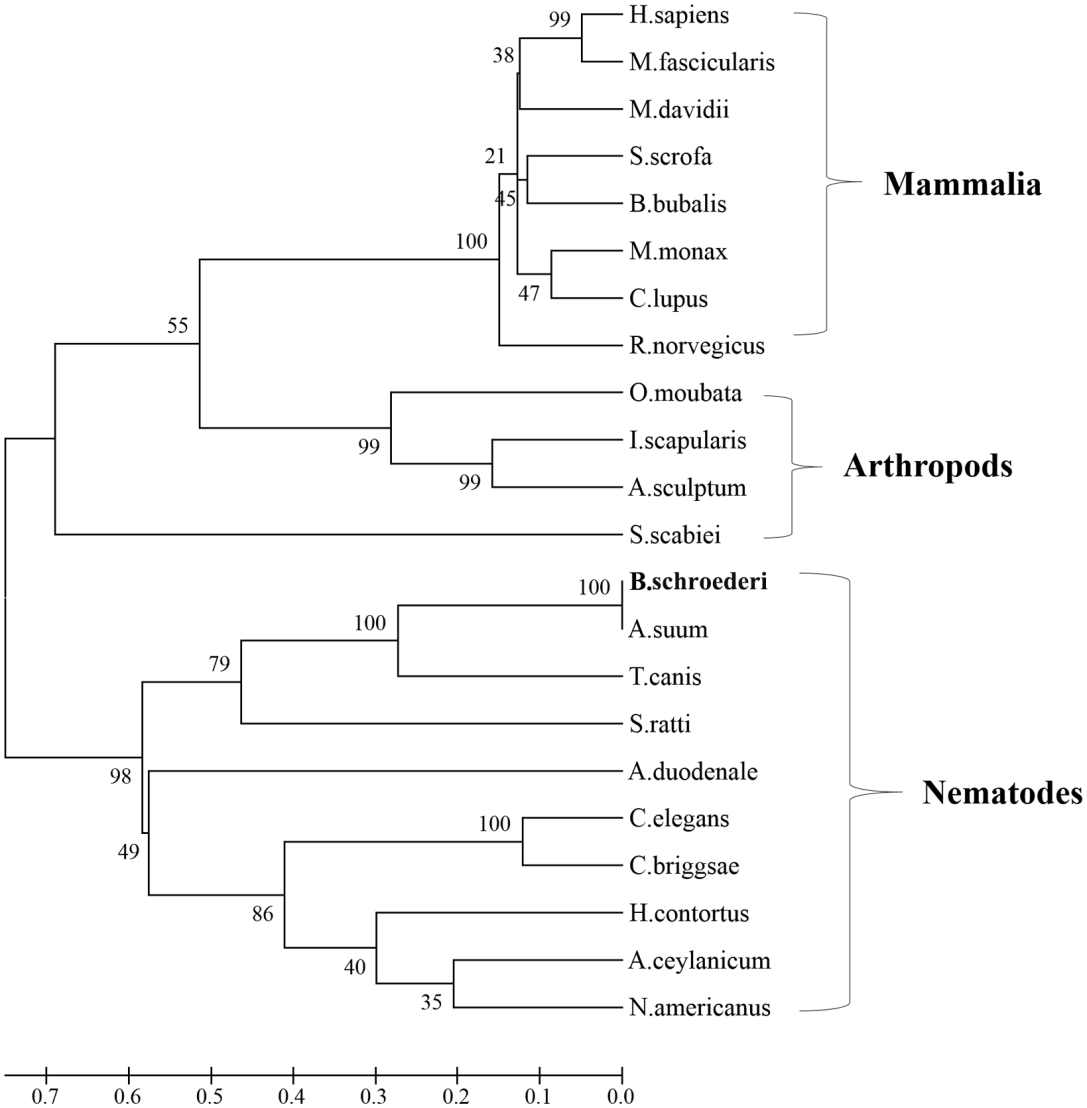
Phylogenetic analysis of GAL proteins. An unrooted phylogenetic tree was inferred by NJ analysis. The scale bar indicates evolutionary distance (0.1 substitutions per position). Nodal support is given in percent (1000 bootstrap replicates). The protein sequences used in the tree are from: *H*. *sapiens* (BAB83625.1), *M*. *fascicularis* (XP_005583254.2), *M*. *davidii* (XP_006764116.1), *S*. *scrofa* (NP_999097.1), *B*. *bubalis* (BAP47874.1), *M*. *monax* (ANI19823.1), *C*. *lupus* (AAS80311.1), *R*. *norvegicus* (NP_037109.1), *O*. *moubata* (AB255165.1), *I*. *scapularis* (XP_002403321.1), *A*. *sculptum* (JAU01832.1), *S*. *scabiei* (KPM04730.1), *A*. *suum* (ERG81589.1), *T*. *canis* (KHN87849.1), *S*. *ratti* (CEF66290.1), *A*. *duodenale* (KIH48405.1), *C*. *elegans* (NP_497215.1), *C*. *briggsae* (XP_002642976.1), *H*. *contortus* (CDJ91991.1), and *N*. *americanus* (XP_013299521.1).

Multiple sequence alignment revealed that Bs-FABP shared the highest similarity (92%) with a homologous protein from *Ascaris suum*, followed by *Toxocara canis* (74%) (Fig 1A). Bs-GAL showed similarity of 72% and 62% with GALs from *A*. *suum* and *T*. *canis* respectively (Fig 1B). Using amino acid sequences of homologs from various species, a phylogenetic tree was constructed in MEGA 5.0 software. The relationship between Bs-FABP and *A*. *suum* FABP was close; the FABPs of nematodes were distant from those of trematodes and cestodes. In addition, the position of Bs-FABP was far from that of FABPs from ectoparasites, especially mites (Fig 2). GAL from *B*. *schroederi* and other nematodes formed one branch of a phylogenetic tree based on that protein; mammals and arthropods (such as scabies mites and ticks) formed separate branches, indicating distant relationships (Fig 3).

**Fig 4:**
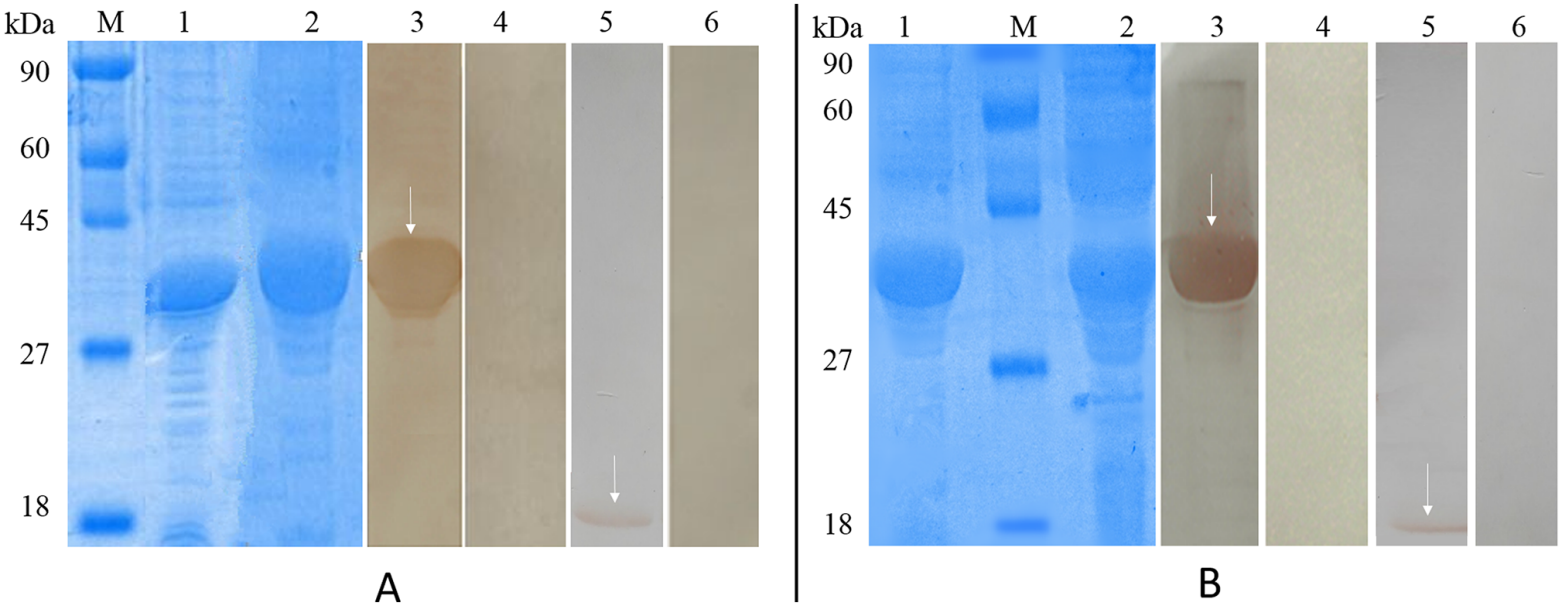
Purification of recombinant *B*. *schroederi* FABP and GAL and western blot analysis. A. M: Protein molecular weight markers; lane 1: Expression of recombinant *B*. *schroederi* FABP (rBs-FABP) in *Escherichia coli*; 2: SDS-PAGE of purified rBs-FABP; 3: western blot of rBs-FABP incubated with positive (infected) panda serum; 4: western blot of rBs-FABP incubated with negative (uninfected) panda serum; 5: western blot of *B*. *schroederi* extracts incubated with rabbit rBs-FABP-IgG; 6: western blot of *B*. *schroederi* extracts incubated with healthy (naïve) rabbit serum. B. M: Protein molecular weight markers; lane 1: SDS-PAGE of purified recombinant *B*. *schroederi* GAL (rBs-GAL); 2: Expression of rBs-GAL in *E*. *coli*; 3: western blot of rBs-GAL incubated with positive (infected) panda serum; 4: western blot of rBs-GAL incubated with negative (uninfected) panda serum; 5: western blot of *B*. *schroederi* extracts incubated with rabbit rBs-GAL-IgG; 6: western blot of *B*. *schroederi* extracts incubated with healthy (naïve) rabbit serum. The white arrows in lanes 3 and 5 indicate the reaction bands of serum and protein.

The genes encoding BsFABP and BsGAL were successfully ligated into expression vector pET-32a(+); plasmids were transformed into *E*. *coli* BL21 and protein expression induced. After 12% SDS-PAGE of the purified protein products, single bands of about 34 kDa were observed for each protein (Fig 4A and 4B), which was in accordance with the expected size (including about 18 kDa for each protein from the pET32a(+) vector).

### Immunoblotting and immunolocalization

Immunoblotting showed that rBsFABP and rBsGAL could be recognized by positive serum from giant pandas and the single band on the membranes indicated good immunogenicity of these recombinant proteins. Crude proteins extracted from *B*. *schroederi* were probed by rabbit rBs-FABP-IgG and rBs-GAL-IgG, resulting in a single band for each, corresponding to the natural Bs-FABP and Bs-GAL (16.7 kDa and 16.1 kDa, respectively), which confirmed that the recombinant proteins were rBs-FABP and rBs-GAL (Fig 4A and 4B).

**Fig 5:**
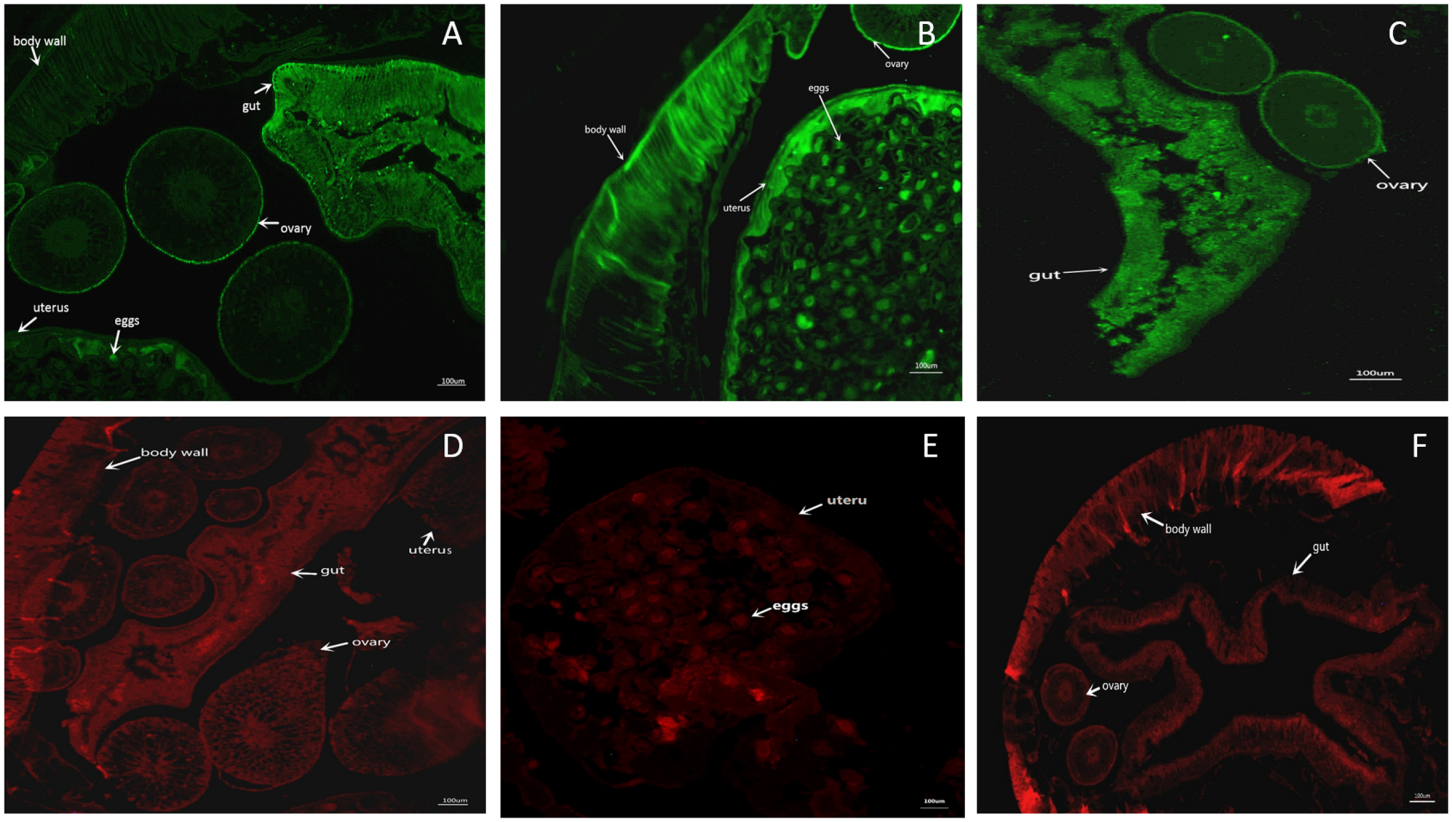
Immunolocalization of Bs-FABP and Bs-GAL proteins in adult female *B*. *schroederi*. Green fluorescence shows the location of Bs-FABP or Bs-GAL. A: rBs-FABP-IgG against FABP; D: negative serum against FABP; B, C: rBs-GAL-IgG against GAL; E, F: negative serum against GAL. The magnification of all images is ×100. Arrows indicate specific areas of the parasite: eggs, body wall, gut, ovary, and uterus.

Anatomical localization of Bs-FABP and Bs-GAL was carried out in histological sections of *B*. *schroederi* adult worms by immunofluorescence using the rabbit polyclonal antisera previously generated. As shown in Fig 5, all sections of the adult worms displayed green fluorescence. Bs-FABP was concentrated in the intestinal and ovarian wall (Fig 5A), while Bs-GAL was concentrated in the body wall, uterus and eggs (Fig 5B and 5C). No fluorescence signal was found in the control samples with healthy rabbit serum (Fig 5D, 5E and 5F).

### Indirect ELISA for giant panda

Following the optimization procedure for the iELISA, we selected optimal concentrations for rBs-FABP and rBs-GAL of 1 μg/well and 3 μg/well, respectively; the serum dilution was 1:160 (data in S1 Table). Critical (cut-off) values were calculated depending on the OD_450_ values obtained from tests of 24 negative serum samples. The cut-off values were 0.171 for rBs-FABP and 0.152 for rBs-GAL. Considering the intra- and interassay variability, CVs for the two recombinant proteins were <10%, which indicated that the ELISAs had good reproducibility and stability.

**Fig 6:**
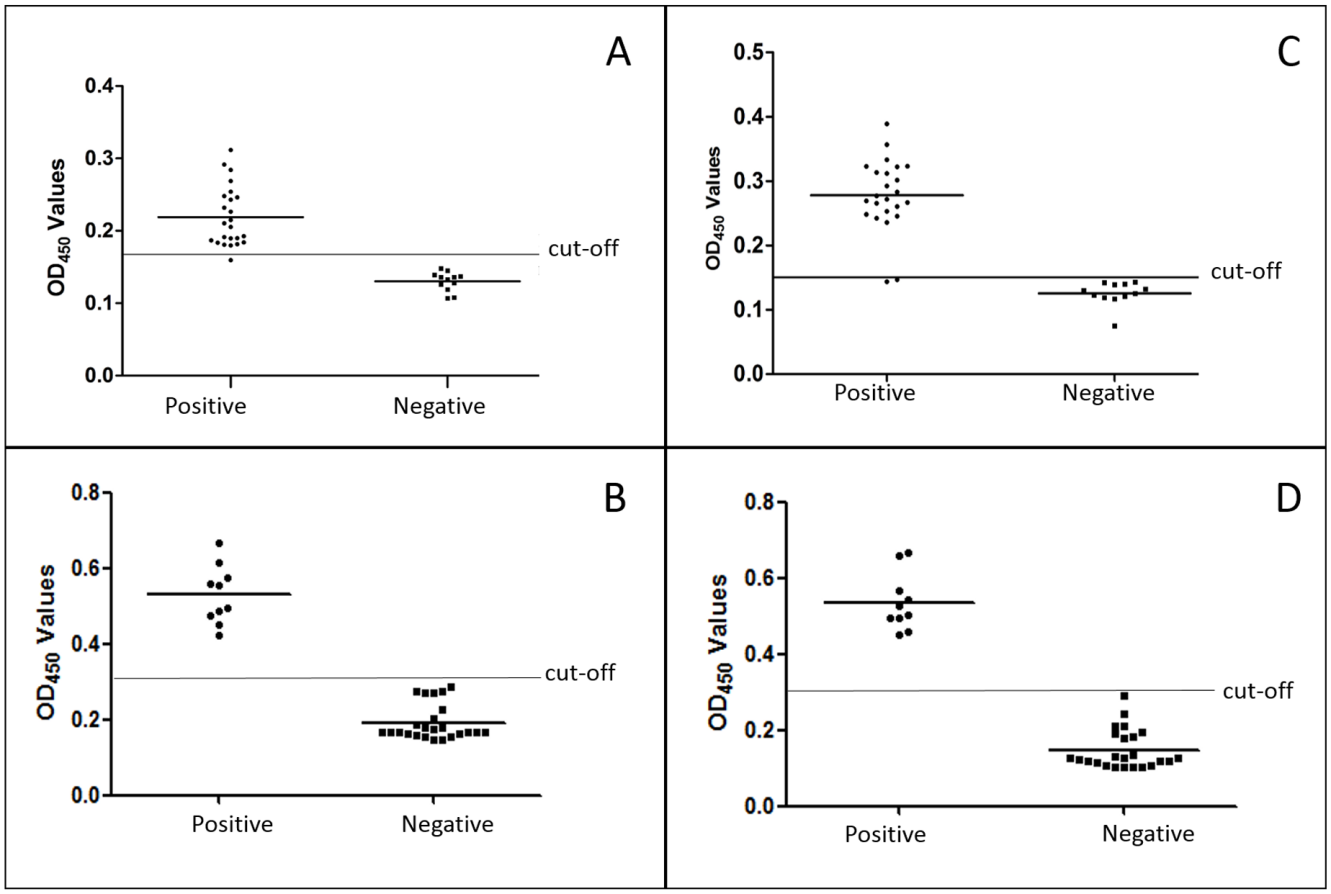
The sensitivity and specificity of indirect ELISAs. The horizontal line of each coordinate axis indicates the cut-off value. A: FABP-coated ELISA with serum of giant pandas. B: FABP-coated ELISA with serum of mice. C: GAL-coated ELISA with serum of giant pandas. D. GAL-coated ELISA with serum of mice.

Twenty-four positive serum samples and 12 negative samples were detected by rBs-FABP and rBs-GAL coated plates. For rBs-FABP, 23 OD_450_ values for positive serum were >0.171 (i.e. the cut-off for the FABP ELISA), and all negative samples were below this value, resulting in a sensitivity of 95.8% (23/24) and specificity of 100% (12/12) (Fig 6A). For rBs-GAL, 22 OD_450_ values of positive serum were >0.152 (the cut-off), and all negative samples were below this value; thus, the sensitivity was 91.7% (22/24) and specificity was 100% (12/12) (Fig 6C).

### Indirect ELISA for mice

Mouse serum was collected after challenge with *B*. *schroederi* eggs for 2 weeks. The titer reached 1:8, displayed by agar diffusion. The iELISA results revealed cut-off values of 0.304 for rBsFABP and 0.333 for rBsGAL, based on 24 negative mouse serum samples. All positive serum samples showed a higher OD_450_ value than their corresponding cut-off, which meant that the sensitivity of the iELISA for both recombinant proteins was 100% (10/10) (Fig 6B and 6D).

## Discussion

Fatty acid-binding proteins are involved in host lipid-mediated immune and tissue differentiation processes, exhibit immunogenicity during immune response, and serve as a molecular target for allergic antibodies [13]. Intestinal parasites can transport fatty acids from the host body for energy metabolism and self-regulation [22]. Galectins are significant immunomodulatory molecules produced by parasites; they play crucial roles in cell adhesion, apoptosis, inflammatory response, and many other physiological and pathological processes [23]. They have been of recent interest to researchers, especially in terms of regulation of host immune-related responses, including in *Plasmodium* [24], *Leishmania* [25], *Trypanosoma cruzi* [26], *Toxoplasma gondii*, and *Schistosoma mansoni* [27].

In this study, Bs-FABP and Bs-GAL were successfully expressed in an *E*. *coli* pET32a(+)-based expression system. The results of immunoblotting showed that rBs-FABP and rBs-GAL could react with sera from giant pandas naturally infected with *B*. *schroederi*, indicating that those positive sera contained specific antibodies against these two proteins. In other words, the recombinant BsFABP and BsGAL had immunoreactivity and could be candidate antigens for the diagnosis of ascariasis.

Based on immunolocalization in adult worms, FABP was distributed in intestinal, reproductive and other tissues where fatty acids are necessary, indicating they could provide energy for the parasite. Appearance of the protein in eggs in the womb demonstrated that energy from fatty acid oxidation may be very important for growth, development and even survival after the eggs are discharged to the outer environment [21]. FABPs transport fatty acids from the host, while parasitic helminths cannot synthesize some fatty acids themselves [28], making FABPs necessary throughout the *B*. *schroederi* body.

GAL was enriched on the surface of *B*. *schroederi*, and this distribution was consistent with reports in other nematodes. For example, GAL of *Dirofilaria immitis* was identified as one of the most abundant proteins in the stratum corneum [29], showing GAL proteins have a significant role in the parasitization process.

Previous methods such as fecal sedimentation-floatation and PCR do not allow early diagnosis of *B*. *schroederi*, so we established iELISAs to evaluate the diagnostic potential of rBs-FABP and rBs-GAL. The sensitivity and specificity were respectively 95.8% and 100% for FABP, and 91.7% and 100% for GAL. Although the sensitivity did not reach 100%, this method still has advantages over other methods, including easier sample collection and earlier detection. After all, this study was a preliminary assessment of ELISA application for diagnosis of giant panda ascariasis and the method will be further developed to make it useful in the field.

Cross-reactivity was not studied in this work. *B*. *schroederi* is the only worm found in captive giant pandas [30], and ectoparasites are rare. Moreover, phylogenetic analysis (Figs 2 and 3) showed that Bs-FABP and Bs-GAL could be clearly distinguished from the related proteins of mites, indicating that there are significant differences between them. In view of these considerations, the ELISA established using rBs-FABP and rBs-GAL has a low probability of cross-reactivity in detection in giant pandas.

It is worth mentioning that the sensitivity and specificity of the ELISAs established here were reasonable, but the OD measurements, especially the positive serum OD_450_ values, were low (significantly <1). This phenomenon differed from the detection of recombinant antigens from other parasites by ELISA. For example, an iELISA for *A*. *suum* showed positive serum OD_450_ values of ≥1 [31, 32]. Therefore, we conducted ELISAs on samples from mice infected with *B*. *schroederi* to further assess the assays. Ten positive samples gave positive results in the ELISAs and significantly higher OD_450_ values than those from giant pandas, revealing that the indirect ELISAs using rBs-FABP and rBs-GAL antigens were of potential diagnostic value.

We hypothesize that the low OD_450_ value in the panda samples might be related to specific characteristics of giant pandas: these animals have some unusual biological characteristics, such as a restricted diet and low reproduction rate; in addition, they have a unique position in evolution [33]. The peculiar biology of giant pandas may also extend to their immune system. Canine distemper virus and canine parvovirus (CPV) are serious viruses for giant pandas, which often lead a high mortality rate [34, 35]. According to one serologic survey of giant pandas and domestic dogs and cats, in animals naturally infected with CPV, the antibody titers in giant pandas were lower than those in dogs and cats [36]. Another report showed that when given multivalent canine distemper attenuated live vaccines specialized for dogs, the antibody titers in giant pandas were low and short-lived; some did not show-up only 3 months after injection [36], whereas in dogs, these vaccines could stimulate the animals to produce enough antibodies to achieve protective efficacy [37]. These phenomena support our hypothesis that there is a distinct immune system/response in giant pandas.

Currently, artificial infection in giant pandas—which are rare and vulnerable animals—is impracticable, studies of their immune mechanism are limited, and test reports on serological diagnosis are unavailable. More follow-up studies are needed to explore reasons for the low titer of giant pandas after injection with vaccines or infection with *B*. *schroederi*.

### Conclusions

We cloned and expressed Bs-FABP and Bs-GAL based on the *B*. *schroederi* transcriptome. The recombinant proteins had good immunogenicity and were widely distributed in adult worms, showing their participation in many biological processes of the parasite. Although the ELISAs established with both rBsFABP and rBsGAL as antigens showed good sensitivity and specificity, rBs-FABP was more sensitive than rBs-GAL, and is thus may be more suitable as a diagnostic antigen in the detection of ascariasis. This trial only included a preliminary evaluation of the diagnostic potential of the two recombinant proteins, but provides support for further study into early diagnosis of *B*. *schroederi* in giant pandas. More clinical tests and screening of other suitable antigens remain necessary.

## Supporting information

S1 Table Determination of optimal antigen concentrations and serum dilution. (DOCX)
