# Supplementary material for: Fatty-binding protein and galectin of Baylisascaris schroederi: Prokaryotic expression and preliminary evaluation of serodiagnostic potential
Source: PLoS One. 2017 Jul 27;12(7):e0182094. doi: 10.1371/journal.pone.0182094 (PMC5531546; doi:10.1371/journal.pone.0182094)
Supplement: S1 Table — (DOCX) [file pone.0182094.s001.docx]

**S1 Table A. Determination of the optimal antigen concentration and serum dilution for rBsFABP**

| Concentration | | Serum dilution | | | | | |
| --- | --- | --- | --- | --- | --- | --- | --- |
|  |  | 1:20 | 1:40 | 1:80 | 1:160 | 1:320 | 1:640 |
| 6μg/well | P  N  P/N | 0.629  0.333  1.889 | 0.576  0.250  2.304 | 0.507  0.175  2.897 | 0.459  0.154  2.981 | 0.410  0.123  3.333 | 0.271  0.129  2.101 |
| 3μg/well | P  N  P/N | 0.622  0.314  1.981 | 0.557  0.237  2.350 | 0.499  0.171  2.918 | **0.444**  **0.141**  **3.149** | 0.372  0.115  3.235 | 0.325  0.104  3.125 |
| 1.5μg/well | P  N  P/N | 0.576  0.301  1.914 | 0.516  0.216  2.389 | 0.475  0.159  2.987 | 0.419  0.138  3.036 | 0.353  0.115  3.070 | 0.313  0.104  3.010 |
| 0.75μg/well | P  N  P/N | 0.509  0.248  2.052 | 0.431  0.184  2.342 | 0.388  0.149  2.604 | 0.356  0.124  2.871 | 0.299  0.114  2.623 | 0.263  0.100  2.630 |
| 0.375μg/well | P  N  P/N | 0.334  0.180  1.856 | 0.312  0.141  2.213 | 0.266  0.123  2.163 | 0.234  0.111  2.108 | 0.198  0.098  2.020 | 0.169  0.096  1.760 |
| 0.188μg/well | P  N  P/N | 0.190  0.137  1.387 | 0.172  0.109  1.578 | 0.163  0.105  1.552 | 0.139  0.092  1.511 | 0.129  0.099  1.303 | 0.132  0.088  1.500 |
| 0.094μg/well | P  N  P/N | 0.125  0.121  1.033 | 0.109  0.108  1.009 | 0.103  0.094  1.096 | 0.116  0.089  1.303 | 0.101  0.090  1.122 | 0.109  0.086  1.267 |
| 0.047μg/well | P  N  P/N | 0.118  0.129  0.915 | 0.105  0.122  0.861 | 0.097  0.103  0.942 | 0.092  0.104  0.885 | 0.099  0.112  0.884 | 0.102  0.095  1.074 |

P:positive serum N:negative serum

**S1 Table B. Determination of the optimal antigen concentration and serum dilution for rBsGAL**

| Concentration | | Serum dilution | | | | | |
| --- | --- | --- | --- | --- | --- | --- | --- |
|  |  | 1:20 | 1:40 | 1:80 | 1:160 | 1:320 | 1:640 |
| 4μg/well | P  N  P/N | 0.481  0.291  1.653 | 0.447  0.256  1.746 | 0.398  0.164  2.427 | 0.348  0.131  2.656 | 0.300  0.109  2.752 | 0.277  0.102  2.716 |
| 2μg/well | P  N  P/N | 0.483  0.320  1.509 | 0.468  0.203  2.305 | 0.407  0.150  2.713 | 0.340  0.122  2.787 | 0.292  0.101  2.891 | 0.251  0.105  2.390 |
| 1μg/well | P  N  P/N | 0.531  0.279  1.903 | 0.458  0.214  2.140 | 0.428  0.152  2.816 | **0.387**  **0.123**  **3.146** | 0.310  0.111  2.793 | 0.228  0.111  2.054 |
| 0.5μg/well | P  N  P/N | 0.368  0.186  1.978 | 0.327  0.146  2.240 | 0.316  0.120  2.633 | 0.267  0.111  2.405 | 0.228  0.101  2.257 | 0.146  0.092  1.587 |
| 0.25μg/well | P  N  P/N | 0.207  0.139  1.489 | 0.195  0.108  1.806 | 0.188  0.106  1.774 | 0.164  0.092  1.783 | 0.139  0.091  1.527 | 0.127  0.092  1.380 |
| 0.125μg/well | P  N  P/N | 0.154  0.124  1.242 | 0.136  0.106  1.283 | 0.121  0.093  1.301 | 0.127  0.088  1.443 | 0.107  0.086  1.244 | 0.108  0.081  1.333 |
| 0.063μg/well | P  N  P/N | 0.107  0.114  0.939 | 0.110  0.096  1.146 | 0.098  0.084  1.167 | 0.096  0.086  1.116 | 0.094  0.087  1.080 | 0.100  0.086  1.163 |
| 0.031μg/well | P  N  P/N | 0.099  0.116  0.853 | 0.097  0.105  0.924 | 0.103  0.095  1.084 | 0.104  0.106  0.981 | 0.101  0.086  1.174 | 0.118  0.088  1.341 |

P:positive serum N:negative serum
